# Patient and system factors associated with unassisted and injurious falls in hospitals: an observational study

**DOI:** 10.1186/s12877-019-1368-8

**Published:** 2019-12-11

**Authors:** Dawn M. Venema, Anne M. Skinner, Regina Nailon, Deborah Conley, Robin High, Katherine J. Jones

**Affiliations:** 1 Division of Physical Therapy Education, College of Allied Health Professions, University of Nebraska Medical Center, 984420 Nebraska Medical Center, Omaha, NE 68198-4420 USA; 2 Allied Health Research Administration, College of Allied Health Professions, University of Nebraska Medical Center, 984000 Nebraska Medical Center, Omaha, NE 68198-4000 USA; 3 CDC Grants Office, Nebraska Medical Center, 987556 Nebraska Medical Center, Omaha, NE 68198-7556 USA; 4 Patient Care Administration, Geriatrics, Methodist Hospital, 8303 Dodge St, Omaha, NE 68114 USA; 5 Department of Biostatistics, College of Public Health, University of Nebraska Medical Center, 984375 Nebraska Medical Center, Omaha, NE 68198-4375 USA

**Keywords:** Hospitals, Accidental falls, Patient safety

## Abstract

**Background:**

Unassisted falls are more likely to result in injury than assisted falls. However, little is known about risk factors for falling unassisted. Furthermore, rural hospitals, which care for a high proportion of older adults, are underrepresented in research on hospital falls. This study identified risk factors for unassisted and injurious falls in rural hospitals.

**Methods:**

Seventeen hospitals reported 353 falls over 2 years. We categorized falls by type (assisted vs. unassisted) and outcome (injurious vs. non-injurious). We used multivariate logistic regression to determine factors that predicted fall type and outcome.

**Results:**

With all other factors being equal, the odds of falling unassisted were 2.55 times greater for a patient aged ≥65 than < 65 (95% confidence interval [CI] = 1.30–5.03), 3.70 times greater for a patient with cognitive impairment than without (95% CI = 2.06–6.63), and 6.97 times greater if a gait belt was not identified as an intervention for a patient than if it was identified (95% CI = 3.75–12.94). With all other factors being equal, the odds of an injurious fall were 2.55 times greater for a patient aged ≥65 than < 65 (95% CI = 1.32–4.94), 2.48 times greater if a fall occurred in the bathroom vs. other locations (95% CI = 1.41–4.36), and 3.65 times greater if the fall occurred when hands-on assistance was provided without a gait belt, compared to hands-on assistance with a gait belt (95% CI = 1.34–9.97).

**Conclusions:**

Many factors associated with unassisted or injurious falls in rural hospitals were consistent with research conducted in larger facilities. A novel finding is that identifying a gait belt as an intervention decreased the odds of patients falling unassisted. Additionally, using a gait belt during an assisted fall decreased the odds of injury. We expanded upon other research that found an association between assistance during falls and injury by discovering that the manner in which a fall is assisted is an important consideration for risk mitigation.

## Background

Up to 1 million hospitalized patients fall annually in the United States [1]. National benchmarks indicate a rate of 3.44 falls/1000 patient days on general medical, surgical, and medical-surgical units [2]. Approximately one-fourth of inpatient falls are injurious [3], with estimated costs exceeding $7000 per injury [4]. Injurious falls are one of 14 hospital-acquired conditions for which hospitals are not reimbursed under the inpatient prospective payment system [5]. Regardless of injury, patients who fall often require a greater length of stay [6–8], are more frequently discharged to a nursing home [7], and may restrict activity due to fear of falling [9]. Risk of falling while hospitalized increases with age [7, 10, 11], as does risk of fall-related injury [12, 13]. Given the aging population [14] and the negative impact of falls, reducing the incidence of falls and fall-related injury is a major focus of patient safety and quality improvement efforts.

Several studies have identified factors associated with inpatient falls [2, 3, 7, 11, 12, 15–22], such as impaired mobility or cognition [7, 17, 20], unit type [2, 3, 12, 20, 21], and staffing characteristics [17, 18, 21, 22]. Further, several studies have identified factors associated with fall-related injury [2, 3, 12, 13, 15, 16, 23]; for example whether or not a fall was assisted [2, 13], and lack of interprofessional teamwork [16]. Many of these studies were conducted in large urban hospitals [7, 11, 12, 15, 17, 20, 23] or used data from the National Database of Nursing Quality Indicators (NDNQI) [2, 3, 18, 21, 22] with samples that underrepresented small hospitals [18, 21] or reported results based on dichotomized bed-size categories of greater or less than 300 beds [2, 22]. Few studies have described risk factors for falls and fall-related injury in rural hospitals [13, 19], and specifically critical access hospitals (CAHs) [16]. CAHs are licensed for up to 25 beds, are located at least 35 miles from another hospital with some exceptions, and receive cost-based reimbursement from the Centers for Medicare and Medicaid Services [24]. CAHs less frequently report or benchmark falls to external organizations [16], despite the value that these processes may provide for quality improvement [25]. CAHs are also less likely than larger hospitals to use a standard fall definition [16]. Therefore, less is known about risk of falls and fall-related injury in rural hospitals including CAHs, compared to larger hospitals.

Because every fall has potential for injury, many hospitals aim to prevent all falls. However, a goal of preventing all falls may incentivize underreporting of non-injurious and assisted falls and discourage patient mobilization [2, 26, 27]. An assisted fall is one in which hospital staff is present and able to control a patient’s descent to the ground [28]. A more appropriate goal than preventing all falls may be to specifically prevent unassisted falls [2], which are more likely to be injurious than assisted falls [2, 13]. Despite numerous studies identifying risk factors for falls in general (without assistance differentiated), and the risk of injury with unassisted falls, we know of only one research group who has studied risk factors for falling unassisted [2, 21, 22]. The focus of two of those studies was on nurse staffing [21, 22], rather than factors more readily modifiable by staff providing direct patient care on a daily basis. Further, those studies that found unassisted falls were more likely to be injurious did not address the method of *how* a fall may be best assisted [2, 13]. The fields of physical and occupational therapy emphasize techniques to keep both patients and staff safe during patient mobilization, including the use of tools such as gait belts to assist patients with mobility [29–31]. As such, staff from those professions may be well suited to collaborate with others in increasing the proportion of hospital falls that are assisted, and therefore less likely to be injurious.

The purpose of this study was to describe fall events and identify risk factors for unassisted and injurious falls in rural hospitals. We report patient and system factors that increase the likelihood of (1) whether the fall was unassisted and (2) whether it resulted in injury. Identification of risk factors for unassisted falls and fall-related injury may guide interventions to reduce unassisted and injurious fall rates.

## Methods

### Design and setting

We conducted an observational analysis of 353 fall events reported by 17 rural hospitals in Nebraska from August 1, 2012 through July 31, 2014. Sixteen of the 17 hospitals were CAHs. The average number of licensed beds was 25.2 ± 6.1. According to the 2010 United States Census, 18.7% of the population of the counties served by the hospitals in this study was ≥65 years of age as compared to 12.7% of the population of the United States [32].

The hospitals reported fall events as part of a research demonstration and dissemination project funded by the Agency for Healthcare Research and Quality (AHRQ). The purpose of this funding mechanism was to: (1) implement safe practices that demonstrate evidence of reducing medical errors, risks, and harms associated with the process of healthcare and (2) inform AHRQ, providers, patients, and payers about implementation of safe practices in diverse settings. Our project, Collaboration and Proactive Teamwork Used to Reduce (CAPTURE) Falls [33, 34], sought to decrease risk of falls and fall-related injury in rural hospitals by implementing a multi-team system [35] to address fall-risk reduction. Each hospital’s multi-team system was led by an interprofessional fall-risk-reduction team that coordinated their fall-risk-reduction program and reported fall-event data to us.

### Variables and measurements

A fall was defined as “a sudden, unintended, uncontrolled downward displacement of a patient’s body to the ground or other object” [36]. This definition included unassisted and assisted falls (when a patient is assisted to the ground by staff). Our definitions for assisted falls and levels of injury were consistent with those used by the NDNQI [28]. For analysis, falls were categorized according to type (unassisted or assisted) and outcome (non-injurious or injurious). Injury included minor harm such as a hematoma, moderate harm such as the need for sutures, major harm such as fracture, or death.

We developed a standard reporting form (Additional File 1) to collect data about fall events. In addition to categorizing fall events according to type and outcome as described above, other data collected using our reporting form were patient factors, such as age and gender, or system factors, such as when the patient was last assessed and alarm usage. Many of our data elements regarding fall events were from the AHRQ Common Formats [36]. We also added fields to record whether a gait belt was used during an assisted fall, and the patient medical record number for tracking repeat falls for a patient within the same admission.

We educated each hospital’s fall-risk-reduction team regarding the rationale for collecting all data elements on the reporting form to improve reliability and validity of the data. This education occurred during Fall 2012 during site visits to each hospital followed by a conference call with all hospitals. As falls occurred, hospitals returned completed reporting forms to the research team via encrypted email or US mail, and data were entered into a Microsoft® Access database. We clarified inconsistencies and missing data in fall-event reports with each fall-risk-reduction team. We provided feedback about accuracy and completeness of reporting during quarterly conference calls with each fall-risk-reduction team throughout the study. Lastly, two members of the research team (DV and KJ) verified fall-event data entered into the database were internally consistent with the description of the fall and externally consistent with our definitions for fall type and outcome.

To calculate fall rates, we requested patient days for acute, skilled, and hospice patients, and hours patients were under observation from each hospital. These data were collected for 2013 and through the end of the project. Patient days was the sum of acute, skilled, and hospice days plus observation hours divided by 24. Total, unassisted, and injurious fall rates were expressed per 1000 patient days. Total falls included assisted, unassisted, injurious, and non-injurious falls.

### Statistical analysis

Data used for statistical analysis are provided in Additional File 2. We used SAS/STAT software from SAS, Version 9.3 (SAS Institute Inc., Cary, NC, USA) for all analyses. We used the Pearson Chi-Square Test or Exact Pearson Chi-Square Test to determine the bivariate association between patient and system factors and fall type and fall outcome. Statistical significance was set at α ≤ .05. We used univariate logistic regression to determine significant patient or system predictors of fall type and outcome. We considered all falls as independent system events not nested by patient because of relatively small numbers of patients with ≥2 falls and the different situations in which repeat falls occurred. We used multivariate logistic regression to determine which patient or system factors best predicted fall type and outcome. We entered any variable that had a *p* value < .15 in the univariate analyses into the multivariate analyses on an exploratory basis to adjust the outcome for the presence of these variables in the model. We did not control for nesting of observations within hospitals because variation between hospitals was too small for the estimation procedure to converge to a solution.

## Results

Three hundred sixteen patients accounted for 353 fall events reported during the study. Most were older adults with respiratory, orthopedic, or cardiovascular diagnoses (Table 1). Fall type was specified for all 353 falls, while fall outcome was specified for 352 falls. Of the 353 falls, 90 (25.5%) were assisted. Of the 352 falls with specified injury level, 113 (32.1%) were injurious. Aggregate fall rates for the study period were 4.0 total, 3.0 unassisted, and 1.3 injurious falls per 1000 patient days.

**Table 1:** Patient Demographic Data^*^

Age of fallers, median (range) in years^†^	77 (19 to ≥90)
Gender (% male)	44.9
Bed Type (%)	
Acute	68.0
Hospice	2.2
Skilled	25.9
Observation	3.5
Diagnostic Category (%)^‡^	
Respiratory	19.9
Orthopedic	17.8
Cardiovascular	15.8
Weakness	12.3
Infection	11.6

^*^Based on 316 unique patients

^†^Specific age was not collected on the reporting form for patients ≥90 years for patient confidentiality

^‡^Diagnostic category was assigned by 2 members of the research team (DV and KJ) based on the diagnoses written on the reporting form by the hospitals. Diagnosis was missing for 24 patients, thus percentages are based on 292 patients with known diagnoses. The top five diagnostic categories are reported. Some patients had more than one diagnosis indicated on the reporting form and therefore contribute towards percentages in more than one category

Table 2 reports bivariate associations between patient factors and fall type and outcome. Fall type was significantly associated with cognitive impairment (*p* < .001) and fall time (*p* = .048). Specifically, a higher proportion of unassisted falls occurred among patients with cognitive impairment and during 10:00 pm to 3:59 am. Fall outcome was significantly associated with age category (*p* = .02), fall location (*p* = .002), and toileting (*p* = .02). Specifically, the proportion of injurious falls increased with age, if the fall occurred in the bathroom, and if the fall was related to toileting, a variable that included factors beyond the patient simply being in the bathroom (see footnotes for Table 2).

**Table 2:** Association Between Patient Factors, Fall Type, and Fall Outcome

		Fall Type (*n* = 353)			Fall Outcome (*n* = 352)^*^		
Patient Factor	Level	Assisted, n (%)	Unassisted, n (%)	*p* value^†^	Non-Injurious, n (%)	Injurious, n (%)	*p* value^†^
Age Category	81+	31 (21.2)	115 (78.8)	.053	90 (61.6)	56 (38.4)	.02
	65–80	29 (23.8)	93 (76.2)		82 (67.2)	40 (32.8)	
	19–64	30 (35.3)	55 (64.7)		67 (79.8)	17 (20.2)	
Gender	Male	34 (21.4)	125 (78.6)	.11	105 (66.5)	53 (33.5)	.60
	Female	56 (28.9)	138 (71.1)		134 (69.1)	60 (30.9)	
Cognitively Impaired^‡^	No	57 (38.3)	92 (61.7)	< .001	101 (68.2)	47 (31.8)	.91
	Yes	33 (16.2)	171 (83.8)		138 (67.6)	66 (32.4)	
Weak^§^	No	36 (23.2)	119 (76.8)	.39	107 (69.5)	47 (30.5)	.58
	Yes	54 (27.3)	144 (72.7)		132 (66.7)	66 (33.3)	
Incontinent^§^	No	84 (26.7)	231 (73.3)	.15	215 (68.5)	99 (31.5)	.51
	Yes	6 (15.8)	32 (84.2)		24 (63.2)	14 (36.8)	
Anti-coagulant^§^	No	87 (26.4)	242 (73.6)	.15	219 (66.8)	109 (33.2)	.11
	Yes	3 (12.5)	21 (87.5)		20 (83.3)	4 (16.7)	
Medications Increase Fall Risk^ǁ^	No	7 (20.0)	28 (80.0)	.39	24 (68.6)	11 (31.4)	.88
	Yes	73 (26.8)	199 (73.2)		183 (67.3)	89 (32.7)	
Bed Type	Not Skilled^¶^	59 (22.9)	199 (77.1)	.054	174 (67.7)	83 (32.3)	.95
	Skilled	31 (33.0)	63 (67.0)		64 (68.1)	30 (31.9)	
Bed Type	Acute	55 (23.3)	181 (76.7)	.10	164 (69.8)	71 (30.2)	.12
	Hospice	0 (0.0)	8 (100.0)		3 (37.5)	5 (65.2)	
	Skilled	31 (33.0)	63 (67.0)		64 (68.1)	30 (31.9)	
	Observation	4 (28.6)	10 (71.4)		7 (50.0)	7 (50.0)	
Fall Time	22:00–03:59	11 (14.1)	67 (85.9)	.048	52 (66.7)	26 (33.3)	.57
	04:00–09:59	28 (32.2)	59 (67.8)		63 (72.4)	24 (27.6)	
	10:00–15:59	24 (25.3)	71 (74.7)		66 (69.5)	29 (30.5)	
	16:00–21:59	25 (28.7)	62 (71.3)		54 (62.8)	32 (37.2)	
Fall Location	In Bathroom	18 (23.7)	58 (76.3)	.68	40 (53.3)	35 (46.7)	.002
	Not in Bathroom	72 (26.0)	205 (74.0)		199 (71.8)	78 (28.2)	
Fall Related to Toileting^#^	No	45 (22.2)	158 (77.8)	.10	148 (72.9)	55 (27.1)	.02
	Yes	45 (30.0)	105 (70.0)		91 (61.1)	58 (38.9)	

^*^Injury was unspecified for one fall

^†^*p* value calculated using Pearson Chi-Square Test or Exact Pearson Chi-Square Test

^‡^Includes the three contributing patient factors of “cognitive impairment,” “impulsive behavior,” or “overestimated ability”

^§^Identified as a contributing patient factor on the reporting form

^ǁ^At the time of the fall, the patient was deemed to be on medication known to increase the risk of fall

^¶^Includes patients in acute, hospice, and observation beds at the time of the fall

^#^Fall was categorized as being related to toileting if the hospital checked “toileting/on commode w/assistance,” “toileting/on commode w/o assistance,” “ambulating to bathroom w/assistance,” “ambulating to bathroom w/o assistance,” or “dressing/undressing related to toileting,” in response to the question “Prior to the fall, what was the patient doing or trying to do?”

Table 3 reports bivariate associations between system factors and fall type and outcome. Fall type was significantly associated with identifying a gait belt as an intervention (*p* < .001), and location of a patient’s room (*p* = .02). Specifically, the proportion of unassisted falls increased when a gait belt was not identified as a fall-risk-reduction intervention for a given patient (i.e. gait belt use was not in the nursing care plan), and for patients in rooms close to the nurse’s station. Fall outcome was significantly associated with assistance during a fall (*p* = .004), use of alarms (*p* = .048), gait belt usage (*p* = .002), and a toileting schedule (*p* = .048). Specifically, the proportion of injurious falls was higher when falls were unassisted, and when alarms and toileting schedules weren’t used. Additionally, a lower proportion of injurious falls occurred when staff was providing assistance with a gait belt prior to the fall.

**Table 3:** Association Between System Factors, Fall Type, and Fall Outcome

		Fall Type (n = 353)			Fall Outcome^*^ (n = 352)		
System Factor	Level	Assisted, n (%)	Unassisted, n (%)	*p* value^†^	Non-Injurious, n (%)	Injurious, n (%)	*p* value^†^
Last Assessed	< 1 h	63 (26.8)	172 (73.2)	.50	164 (70.1)	70 (29.9)	.77
	1–2 h	10 (26.3)	28 (73.7)		25 (65.8)	13 (34.2)	
	> 2 h	4 (44.5)	5 (55.6)		7 (77.8)	2 (22.2)	
Fall Assisted	No				167 (63.7)	95 (36.3)	.004
	Yes				72 (80.0)	18 (20.0)	
Alarms in Use^‡^	No	43 (22.8)	146 (77.2)	.20	119 (63.3)	69 (36.7)	.048
	Yes	47 (28.7)	117 (71.3)		120 (73.2)	44 (26.8)	
Non Slip Floor Mat^§^	No	88 (25.5)	257 (74.5)	>.99	233 (67.7)	111 (32.3)	.73
	Yes	2 (25.0)	6 (75.0)		6 (75.0)	2 (25.0)	
Low Bed^§^	No	28 (22.0)	99 (78.0)	.27	88 (69.8)	38 (30.2)	.56
	Yes	62 (27.4)	164 (72.6)		151 (66.8)	75 (33.2)	
Patient and Family Education^§^	No	41 (23.3)	135 (76.7)	.34	115 (65.7)	60 (34.3)	.38
	Yes	49 (27.7)	128 (72.3)		124 (70.1)	53 (29.9)	
Gait Belt Identified as Intervention^ǁ^	No	25 (12.4)	177 (87.6)	<.001	138 (68.7)	63 (31.3)	.73
	Yes	65 (43.0)	86 (57.0)		101 (66.9)	50 (33.1)	
Fall Assistance and Gait Belt Usage^¶^	Staff not providing hands on assist prior to fall	2 (0.80)	263 (99.2)	NA	167 (63.3)	97 (36.7)	.002
	Staff providing hands on assist prior to fall but without gait belt	42 (100.0)	0 (0.0)		31 (73.8)	11 (26.2)	
	Staff providing hands on assist prior to fall with gait belt	46 (100.0)	0 (0.0)		41 (89.1)	5 (10.9)	
Sitter^§^	No	88 (25.8)	253 (74.2)	.54	229 (67.4)	111 (32.6)	.35
	Yes	2 (16.7)	10 (83.3)		10 (83.3)	2 (16.7)	
Toileting Schedule^§^	No	65 (23.4)	213 (76.6)	.08	181 (65.3)	96 (34.7)	.048
	Yes	25 (33.3)	50 (66.7)		58 (77.3)	17 (22.7)	
Patient Close to Nurse’s Station^§^	No	73 (28.9)	180 (71.1)	.02	168 (66.7)	84 (33.3)	.43
	Yes	17 (17.0)	83 (83.0)		71 (71.0)	29 (29.0)	
Purposeful Hourly Rounding^§^	No	36 (21.2)	134 (78.8)	.07	110 (64.7)	60 (35.3)	.22
	Yes	54 (29.5)	129 (70.5)		129 (70.9)	53 (29.1)	

^*^Injury was unspecified for one fall

^†^*p* value calculated using Pearson Chi-Square Test or Exact Pearson Chi-Square Test

^‡^“Alarms in Use” was categorized as being in place if the hospital identified either bed or chair alarms as interventions to be used to prevent the reported fall. It does not necessarily mean that an alarm was sounding at the time of the fall

^§^System characteristic deemed to be present if the hospital identified this intervention as in use to prevent the reported fall

^ǁ^“Gait Belt Identified as Intervention” was categorized as being in place if the hospital identified a gait belt as an intervention to be used to prevent the reported fall (i.e. gait belt use was in the nursing care plan). It does not necessarily mean a gait belt was in use at the time of the fall

^¶^“Fall Assistance and Gait Belt Usage” indicates whether hands on assist was being provided at the time of the fall, and whether or not a gait belt was being used to provide that assistance. For the purposes of this system variable, the 2 falls in the “Assisted” column for the row “Staff not providing hands on assist prior to fall” reflect 2 falls in which staff were not providing hands on assist immediately prior to the fall but did provide hands on assist once the patient began to fall

Table 4 provides results of the logistic regression for patient and system factors associated with increased odds of a patient falling unassisted. Considering the adjusted odds ratios specifically, with all other factors being equal, the odds of falling unassisted were: 2.55 times greater for a patient ≥65 than one 19–64 years old (95% confidence interval [CI]: 1.30–5.03), 3.70 times greater for a patient with cognitive impairment than without (95% CI: 2.06–6.63), and nearly 7 times greater if a gait belt was not identified as an intervention for a patient than if it was identified (95% CI: 3.75–12.94).

**Table 4:** Odds Ratios for Patient and System Factors Associated with Increased Odds of Falling Unassisted

Patient or System Factor	Crude Odds Ratio^*^ (95% CI)	Adjusted Odds Ratio^†^ (95% CI)
Age > 65 years^‡^	1.89 (1.11–3.21)	2.55 (1.30–5.03)
Cognitively Impaired^§^	3.21 (1.95–5.28)	3.70 (2.06–6.63)
Gait Belt NOT Identified as an Intervention^ǁ^	5.35 (3.15–9.08)	6.97 (3.75–12.94)

CI, Confidence Interval.

^*^Calculated using univariate logistic regression with all falls considered independent events

^**†**^Calculated using multivariate logistic regression and adjusted for the influence of other variables in the model

^‡^Reference category = 19 to 64 years

^§^Cognitively impaired includes the three contributing patient factors of “cognitive impairment,” “impulsive behavior,” or “overestimated ability.” Reference category = not cognitively impaired

^ǁ^Gait Belt NOT Identified as an Intervention” means the hospital did not identify a gait belt as an intervention to prevent a reported fall (i.e. gait belt use was not in the nursing care plan). Reference category = Gait Belt was Identified as an Intervention to Prevent a Reported Fall

Table 5 provides the results of the logistic regression for patient and system factors associated with increased odds of a patient experiencing fall-related injury. Considering the adjusted odds ratios specifically, with all other factors being equal, the odds of an injurious fall were: 2.55 times greater for a patient ≥65 than one 19–64 years old (95% CI: 1.32–4.94), 2.48 times greater for a fall that occurred in the bathroom compared to one that occurred elsewhere (95% CI: 1.41–4.36), and 3.65 times greater for a fall that occurred when hands-on assist was being provided without a gait belt, compared to one that occurred when hands-on assist with a gait belt was provided (95% CI: 1.34–9.97).

**Table 5:** Odds Ratios for Patient and System Factors Associated with Increased Odds of Fall-Related Injury

Patient or System Factor	Crude Odds Ratio^*^ (95% CI)	Adjusted Odds Ratio^†^ (95% CI)
Age > 65 years^‡^	2.20 (1.22–3.96)	2.55 (1.32–4.94)
Fall in Bathroom^§^	2.23 (1.32–3.77)	2.48 (1.41–4.36)
NO Alarms in Use^ǁ^	1.58 (1.00–2.49)	1.46 (0.89–2.41)
Unassisted^¶^	2.28 (1.28–4.04)	1.48 (0.69–3.14)
Hands on Assist WITHOUT Gait Belt^¶^	4.76 (1.99–14.15)	3.65 (1.34–9.97)

CI, Confidence Interval.

^*^Calculated using univariate logistic regression with all falls considered independent events

^**†**^Calculated using multivariate logistic regression and adjusted for the influence of other variables in the model

^‡^Reference category = 19 to 64 years

^§^Reference category = fall occurred in location other than the bathroom

^ǁ^NO Alarms in Use means the hospital did not report that either bed or chair alarms were in use as interventions to prevent a reported fall. Reference category = either bed or chair alarms were checked as interventions in use to prevent a reported fall

^¶^Unassisted and Hands on Assist WITHOUT Gait Belt indicate whether or not hands on assist was provided at the time of the fall and whether or not a gait belt was used to provide that assistance. Reference category = Hands on Assist WITH Gait Belt

## Discussion

In our study, we determined risk factors that increased the odds of a patient falling unassisted and whether the fall was injurious. We report risk factors for patient falls in rural hospitals, which are underrepresented in the literature [13, 16, 19] and in external benchmarking databases [2, 16, 18, 21, 22]. Furthermore, we complemented prior research that identified risk factors for falling unassisted [2, 21, 22], and a relationship between assistance during falls and injury [2, 13]. Patient factors of increased age and cognitive impairment, and the system factor of not identifying a gait belt as an intervention for a given patient, increased the odds of falling unassisted. The patient factor of increased age, and system factors of a fall occurring in the bathroom and hands-on assist being provided, but without a gait belt, increased the odds of fall-related injury.

### Comparison with other research

Few published studies have reported risk factors for falling unassisted [2, 21, 22]. Staggs et al. [21, 22] used data from the NDNQI to explore the relationship between nurse staffing characteristics and unassisted falls. In one study, unit type, total nurse hours per patient day, skill mix of nursing staff, and average tenure of registered nurses were related to unassisted fall rates [21]. In another study, higher non-registered nurse staffing was associated with higher rates of unassisted falls on all unit types except rehabilitation [22]. In a third study, Staggs et al. [2] used NDNQI data to consider factors besides nurse staffing, and found male gender, being assessed for risk, not having a fall-risk-reduction protocol in place, and unit type increased the odds of unassisted falls. We collected data relative to fall-risk factors found in the AHRQ Common Formats [36]. While some work by Staggs et al. [2, 21, 22] highlighted the relationship between nurse staffing and unassisted falls, these studies did not advance understanding of how processes of care influence unassisted falls. The system factors we considered are evidence-based interventions more readily modifiable on an everyday basis by nurses providing direct patient care than staffing characteristics. Thus, we provide evidence regarding care processes associated with unassisted falls such as identifying when a gait belt is appropriate to use for a given patient.

Considerable research has identified factors that increase risk of fall-related injury [2, 3, 12, 13, 15, 16, 23]. Our findings agree with this research in that increased patient age was associated with increased odds of injury [12, 13], as did falls occurring in the bathroom [13]. Our findings agreed with others in that cognitive impairment was not associated with increased odds of injury [12, 13]. Unlike other researchers [2, 13], we did not find gender to significantly predict injury. Hitcho et al. [15] reported elimination-related falls as the only variable in their multivariate analysis to significantly predict injury. Our factor of “fall related to toileting” was significant only in the bivariate analysis. Mion et al. [23] reported specific medications (e.g. antidepressants, antipsychotics, opiates, and diuretics) increased risk of fall-related injury. We did not find a significant association between medication use and injury, perhaps because most patients in our sample, regardless of injury, were taking medications known to increase fall risk. Lastly, Staggs et al. [2] and Krauss et al. [13] found unassisted falls were associated with increased odds of injury relative to assisted falls. We found being unassisted during a fall was significant only in the bivariate analysis. The critical factor regarding assistance that predicted injury in our multivariate model was whether or not assistance was provided with a gait belt. Our work extends that of others [2, 13] by suggesting assisting a fall without a gait belt results in greater odds of injury than not assisting the fall at all.

Some system factors were significantly related to fall type and outcome in our bivariate analysis (use of alarms, scheduled toileting, and proximity to the nurses’ station), but were not significant in the multivariate analysis. The AHRQ [37] recommends not relying on alarms for hospital fall prevention because previous research found them ineffective [38], which is consistent with our multivariate results. Scheduled toileting, particularly as part of scheduled rounding, is considered a best practice for preventing falls [37]. We found a higher proportion of unassisted falls in patients whose rooms were close to the nurses’ station, contrary to the assumption that staff could respond quickly if a patient mobilized unaided. We discovered many falls that occurred in rooms close to the nurses’ station were repeat falls, and as such, may have occurred in patients at especially high risk for falling. Despite these factors being insignificant in our multivariate model, we do not suggest abandoning these common fall-risk-reduction interventions. Clinical judgment suggests these interventions may help staff be present before or during patient mobility. Absent staff cannot assist a patient with mobility in a safe manner, such as by using a gait belt.

### Practical implications

Our study provides several practical implications for hospital fall-risk-reduction programs. First, we demonstrate that use of gait belts, a simple and inexpensive patient safety tool, reduces the risk of falling unassisted and the risk of fall-related injury. A person is stable when he is able to control his center of mass (the center of one’s total body mass) in relationship to his base of support (the area of one’s body in contact with the supporting surface) [39]. A gait belt is typically applied just above the pelvis, near the center of mass. By holding the gait belt, a healthcare provider can help a patient maintain his center of mass over his base of support. A gait belt can also be used to control a patient’s descent to a lower surface, should he begin to fall [29, 30]. The AHRQ recommends gait belts only for patients with cognitive impairment [37], but we found value in their use among a broad sample of patients. We discovered failure to identify a gait belt as a fall-risk-reduction intervention increased the odds of an unassisted fall. This failure may occur when a patient is not accurately identified as needing hands-on assist with mobility, or when gait belt use is not part of a hospital’s culture. We also found providing hands-on assist without a gait belt increased the odds of fall-related injury relative to providing hands-on assist with a gait belt, surprisingly more so than a fall simply being unassisted. When a gait belt is not used, a healthcare provider may grasp a patient’s arm, potentially leading to injuries such as skin tears or dislocation of the shoulder joint during a fall.

Second, our data support a role for rehabilitation therapists on hospital fall-risk-reduction teams. The use of gait belts and other strategies for safe patient mobilization are emphasized in physical and occupational therapy [29–31]. Although nurses and nursing assistants are trained in the use of gait belts, their training is not as robust in the mechanics of body movement and stability. Also, their daily practice does not involve routine use of gait belts in nearly every patient encounter, as occurs in physical and occupational therapy. Hoyer et al. [31] reported rehabilitation therapists received more training in how to safely mobilize patients than nurses, and nurses felt less confident than therapists in their ability to mobilize patients. Rehabilitation therapists can contribute to fall-risk-reduction teams by sharing their expertise regarding safe patient mobilization of patients. This education can occur when consulting with nursing staff on individual patients in the context of direct patient care, but also more broadly throughout the organization via staff competency training on safe patient mobilization strategies. This potential collaboration between rehabilitation therapists and other staff demonstrates an interprofessional team approach to fall-risk reduction, in which falls are considered a measure of organizational quality rather than nursing quality [16].

Third, staff knowledge of safe patient mobilization strategies may prevent unintended consequences of reduced patient mobility and subsequent functional decline due to an assumption that keeping patients from moving will prevent falls [26, 27, 40]. Growden et al. [27] suggested hospital fall-risk-reduction teams should focus on promoting the positive outcome of safely mobilizing patients rather than preventing the negative outcome of falls. We believe this suggestion relates to the findings that unassisted falls increase the odds of injury [2, 13] and that when a fall is assisted with a gait belt, the odds of injury are decreased. An unassisted fall implies staff did not accurately identify the patient needed assistance, did not reliably implement fall-risk-reduction interventions, or were simply absent when the fall occurred [2, 26]. Assisted falls and unassisted falls should not be viewed equally when considering the quality of a hospital’s fall-risk-reduction program. Rather, an assisted fall should be considered a success [2]. Staff was in the right place at the right time with the tools and knowledge to assist the patient during the fall [2, 21], which again speaks to the opportunity for collaboration between nursing and rehabilitation therapists to develop strategies for safe patient mobilization.

Finally, we demonstrated the benefit of fall-event reporting in rural hospitals by addressing barriers for benchmarking and learning from fall-event data [16]. We needed to standardize the definitions of a fall, an assisted fall, and fall-related injury as prerequisites for analyzing data and calculating valid fall rates. Prior to our project, only 3 of the hospitals used the AHRQ standard fall definition [36], and only 10 used a standard fall definition from any source. Our reporting form and database became a shared resource for organizational learning among hospitals with limited resources for quality improvement and small numbers of fall events from which to learn. We were able to aggregate data to provide a wider range of information on patient and system factors related to falls than was previously available in these hospitals.

### Strengths and limitations

A strength of our study is that we examined risk factors for unassisted and injurious falls in an understudied setting of hospitals with 50 beds or less. Although our findings are specific to rural hospitals in one state, they may apply to other facilities that care for a high proportion of older adults. Our patient sample is slightly older than those of many studies addressing risk factors for falls [2, 12, 13, 15, 23], yet is similar to patients studied by others [7, 20]. A limitation is our sample size is small relative to other studies that investigated factors related to falling unassisted [2, 21, 22] and experiencing fall-related injury [2, 3, 12, 13, 15, 16, 23]. However, many of our findings agree with larger studies [12, 13], our findings about gait belt use complement studies that reported the relationship between injury and assisted falls [2, 13], and the magnitudes of our adjusted odds ratios are quite convincing. A final strength of our study is our effort to ensure reliability of reported data. We educated fall-risk-reduction teams about how to complete the reporting form, provided ongoing feedback, and followed up on missing and/or inconsistent data in fall-event reports.

## Conclusions

This study provides information regarding risk factors associated with unassisted and injurious falls in rural hospitals. We addressed barriers to reporting, aggregating, and benchmarking fall-event data in these hospitals. We expanded upon other research that found an association between assistance during falls and injury by discovering that the manner in which a fall is assisted is an important consideration for risk mitigation. Additional research is needed to determine best practices in assessing and maintaining the competency of non-rehabilitation therapy staff to safely assist patients with mobility.

## Supplementary information

**Additional file 1.** CAPTURE Falls Event Reporting Form. Standardized reporting form (.pdf) used to collect patient and system data about fall events.

**Additional file 2.** CAPTURE_Falls_Data_and_Dictionary. Spreadsheet (.xlsx) of raw data used to conduct the statistical analysis presented within the manuscript. The file includes data from which fall rates were calculated; data for individual fall events, a data dictionary, and look up tables referred to in the data dictionary. In accordance with our Institutional Review Board’s guidance, the patient medical record numbers were replaced with mock values.

## Data Availability

All data generated or analyzed during this study are included in this published article and a supplementary information file (Additional File 2). In accordance with our Institutional Review Board’s guidance, the medical record numbers were replaced with mock values.

## Abbreviations

AHRQ: Agency for Healthcare Research and Quality
CAH: Critical access hospital
CAPTURE Falls: Collaboration and Proactive Teamwork Used to Reduce Falls
CI: Confidence interval
NDNQI: National Database for Nursing Quality Indicators
