# Supplementary material for: Patient and system factors associated with unassisted and injurious falls in hospitals: an observational study
Source: BMC Geriatr. 2019 Dec 11;19:348. doi: 10.1186/s12877-019-1368-8 (PMC6907170; doi:10.1186/s12877-019-1368-8)
Supplement: Supplementary file 1 — Additional file 1. CAPTURE Falls Event Reporting Form. Standardized reporting form (.pdf) used to collect patient and system data about fall events. [file 12877_2019_1368_MOESM1_ESM.pdf]

Report Date: ___________ Completed By: ________________

CAPTURE Falls Event Learning Form

Definition of fall: For the purposes of patient safety, a fall is a sudden, unintended, uncontrolled downward displacement of a patient's body to the ground or other object. This definition includes unassisted falls and assisted falls (i.e., when a patient begins to fall and is assisted to the ground by another person).

1. Patient Medical Record Number: ______________________ 2. Patient Admission date: ___________

3. Admission Type at time of fall: ☐ Acute ☐ Swing ☐ Hospice ☐ Observation ☐ Outpatient ☐ Visitor

4. Patient Age (if older than 90 indicate >90): ______________ 5. Patient Gender: ☐ Male ☐ Female

6. Patient's principal admitting diagnosis: ____

7. Date of Fall: _____________ 7a. Time of Fall (military time): ____________

8. Ambulatory Status Time of Fall: ☐ Not ambulatory ☐ With assist of 2 ☐ With assist of 1 ☐ Independent

9. Where did the fall occur?
- ☐ Inpatient care area
  - ☐ Bedside
  - ☐ Chairside
  - ☐ Bathroom
  - ☐ Hallway
- ☐ Emergency department
- ☐ Therapy area (PT, OT, ST)
- ☐ Radiology/imaging area, including mobile
- ☐ Outside area (i.e., grounds of this facility)
- ☐ Other: Please specify __________________________

10. Did staff assist the patient (hands on) during the fall?

☐ Yes → 10a. Was a gait belt used? ☐ Yes ☐ No ☐ Unknown

☐ No → 10b. Was the fall observed? ☐ Yes, by staff ☐ Yes, by family, visitor or another patient ☐ No

11. If unassisted and not observed, how did staff discover the fall?
- ☐ Patient found on floor
- ☐ Notified by non-clinical staff
- ☐ Reported by patient
- ☐ Alarm sounding
- ☐ Unknown
- ☐ Notified by family/friend/another patient
- ☐ Notified by ancillary care staff
- ☐ Patient calling for help
- ☐ Patient call light
- ☐ Other: Please specify __________________________

12. DESCRIBE THE FALL, how it occurred, where in detail it occurred, how it was discovered (a narrative may be attached):

____

____

____

____

13. What type of injury was sustained? *CHECK ONE, IF MORE THAN ONE, CHECK MOST SEVERE*
- ☐ No Injury, no signs or symptoms resulting from the fall (x-ray, CT scan or other post fall evaluation resulted in finding of no injury)
- ☐ Fracture
- ☐ Dislocation
- ☐ Intracranial injury
- ☐ Skin tear, abrasion, hematoma or significant bruising
- ☐ Laceration requiring sutures or steri-strips
- ☐ Other: Please specify ____

13a. What was the extent of harm to the patient as a result of the fall? *CHECK FIRST OPTION THAT IS APPLICABLE*
- ☐ **Death:** Patient died as a result of injuries sustained from the fall.
- ☐ **Major:** Fall resulted in surgery, casting, traction, consultation for neurological (e.g. skull fracture, subdural hematoma) or internal injury (e.g. rib fracture, liver laceration) or need for blood products.
- ☐ **Moderate:** Fall resulted in suturing, application of steri-strips/skin glue, splinting or muscle/joint strain.
- ☐ **Minor:** Fall resulted in application of dressing, ice, cleaning of wound, limb elevation, topical medication, bruise or abrasion.

14. Which of the following additional treatments or monitoring were performed as a result of the fall?
CHECK ALL THAT APPLY
☐ Transfer, including transfer to higher level care area within facility, transfer to another facility
☐ Monitoring, including observation, physiological examination, laboratory testing, phlebotomy, and/or imaging studies
☐ Medication therapy including change in pre-incident dose
☐ Surgical/procedural intervention
☐ Respiratory support (e.g., ventilation, tracheotomy)
☐ Unknown
☐ Other intervention: Please specify ______________________________

15. Did, or will, the fall result in an increased length of stay? *CHECK ONE*
☐ Yes ☐ No ☐ Unknown

16. Prior to the fall, what was the patient doing or trying to do? *CHECK ONE*
☐ Toileting/on commode w/assistance
☐ Ambulating w/assistance
☐ Ambulating to bathroom w/assistance
☐ Dressing/undressing
☐ Dressing/undressing related to toileting
☐ Transferring w/assistance
☐ Reaching for an item
☐ Rolled out / Slipped off of bed
☐ Geri chair related
☐ Unknown
☐ Toileting/on commode w/o assistance (left alone)
☐ Ambulating w/o assistance
☐ Ambulating to bathroom w/o assistance
☐ Showering
☐ Dressing/undressing related to showering
☐ Transferring w/o assistance
☐ Loss of consciousness
☐ Chair/recliner related
☐ Wheelchair related
☐ Other: Please Specify ____________________

17. Was the patient using an assistive device or other type of equipment at the time of the fall?
☐ Yes → 17a. What was the device or equipment? ________________________________
☐ No
☐ Unknown

18. Prior to the fall, was a fall risk assessment documented? *CHECK ONE*
☐ Yes → 18a. Was the patient determined to be at risk for a fall?
☐ Yes → 18b. What was the patients score on the fall risk assessment? ___________
☐ No
☐ Unknown
☐ No
☐ Unknown

19. Prior to this fall, has the patient fallen while hospitalized? CHECK ALL THAT APPLY
☐ Yes, during this admission
☐ Yes, during a previous admission
☐ No
☐ Unknown

20. Which of the following were in place and being used to prevent falls for this patient?
CHECK ALL THAT APPLY
☐ Alarm - Bed
☐ Alarm - Chair
☐ Assistive devices (e.g., wheelchair, walker, commode)
☐ Bed in low position
☐ Call light/personal items within reach
☐ Change in medication (e.g., timing or dosing)
☐ Gait Belt
☐ Hip and/or joint protectors
☐ Non-slip footwear
☐ Non-slip floor mats
☐ NOT to be left alone while toileting
☐ Orthostatic vital signs monitoring
☐ Patient and family education
☐ Patient placed close to nurses' station
☐ Physical/Occupational therapy includes strengthening; gait, balance, transfer training
☐ Purposeful rounding
☐ Sitter
☐ Supplemental environmental or area lighting
☐ Toileting regimen
☐ Video monitoring
☐ Visible identification of patient as being at risk for fall (e.g., falling star)
☐ Other: Specify__________________________
☐ NONE

21. Which equipment/devices/furniture contributed to the fall?

- ☐ None
- ☐ Alarm, bed
- ☐ Alarm, chair
- ☐ Assistive device (walker, cane, etc)
- ☐ Bed rails
- ☐ Call Light
- ☐ Gait belt
- ☐ Restraints
- ☐ Wheelchair
- ☐ Other: Please specify____________________

→ 21a. How did the equipment device contribute to the fall?

____

____

22. At the time of the fall, was the patient on medication known to increase the risk of fall?

- ☐ Yes →
- ☐ No
- ☐ Unknown

31. Please indicate the number of each routine medication prescribed:

____ Cardiovascular ____ Diuretics ____ Psychotropics

____ Hypnotics ____ Sedatives ____ Analgesics

____ Antihypertensives ____ Laxatives

23. Which **organizational factors** contributed to the event? CHECK ALL THAT APPLY

Environment
- ☐ Culture of safety, management of staff
- ☐ Physical surroundings cluttered
- ☐ Physical surroundings not customized to accommodate pt's mobility limitations

Staff Qualifications
- ☐ Lack of competence (qualifications, experience)
- ☐ Lack of training (use of gait belt, transfers, lifts)

Supervision/support
- ☐ Lack of clinical supervision
- ☐ Lack of managerial supervision
- ☐ Poor teamwork

Policies and procedures, includes clinical protocols
- ☐ Absence of policies
- ☐ Poor clarity of policies
- ☐ Lack of compliance with policies

Information About Fall Risk Status
- ☐ Not Available
- ☐ Not Accurate
- ☐ Not Legible

Communication
- ☐ Supervisor to staff
- ☐ Among staff or team members
- ☐ Staff to patient (or family)
- ☐ Fall associated with a handoff

Human factors (Staff)
- ☐ Fatigue
- ☐ Stress
- ☐ Inattention
- ☐ Cognitive factors
- ☐ Health issues

External factors
- ☐ Family/Visitor involvement

24. Which **patient** factors contributed to the event? CHECK ALL THAT APPLY

- ☐ Dizziness/Vertigo
- ☐ Hypotension
- ☐ Procedure within last 24 hours
- ☐ Constipation
- ☐ Cognitive impairment
- ☐ Impulsive behavior
- ☐ Overestimated ability
- ☐ Neurological Comorbidities (e.g. previous CVA, MS, Parkinson's Disease)
- ☐ Weakness
- ☐ Anticoagulant / bleeding disorder
- ☐ Bowel Prep in Progress
- ☐ Incontinence/urgency
- ☐ Symptomatic depression
- ☐ Sensory Impairment (vision, hearing, balance, etc.)
- ☐ Morbid obesity
- ☐ Other: PLEASE SPECIFY ____________________
